# Avian Reservoirs of the Agent of Human Granulocytic Ehrlichiosis?

**DOI:** 10.3201/eid0812.010527

**Published:** 2002-12

**Authors:** Thomas J. Daniels, Gertrude R. Battaly, Dionysios Liveris, Richard C. Falco, Ira Schwartz

**Affiliations:** * Fordham University, Armonk, New York; † New York Medical College, Valhalla, New York

**To the Editor:** Human granulocytic ehrlichiosis (HGE), first described in 1994 (1), is the second-most common tick-borne disease in the United States; Lyme disease is the most prevalent. Both diseases are transmitted by blacklegged ticks (*Ixodes scapularis* Say) that are abundant in southern New York state (2). Factors that influence the risk for infection, particularly the role of wildlife in transmitting the etiologic agent, *Anaplasma* (formerly *Ehrlichia*) *phagocytophilum* (3,4), to vector ticks are not well understood. In the absence of transovarial transmission (5,6), acquisition of *A. phagocytophilum* by its vector must result either from feeding on the blood of reservoir-competent hosts or by cofeeding of uninfected ticks in close proximity to infected ticks (7).

The role that birds may play in HGE ecology is potentially very important. Birds in the northeastern United States routinely host immature *I. scapularis* (8), and several species are competent reservoirs of *Borrelia burgdorferi* (9), the causative agent of Lyme disease, thus providing an opportunity to transport infected ticks to new areas. While data for HGE are lacking, *A. phagocytophilum–*infected *I. ricinus* nymphs have been collected from migrating birds in Sweden (10); reservoir competence was not established, however. Our goal was to determine if several common bird species might serve as reservoirs of *A. phagocytophilum* and, as such, transmit the pathogen to feeding *I. scapularis* larvae.

Birds were sampled at a deciduous woodland preserve in Tarrytown, Westchester County, New York, an area where Lyme disease and HGE are endemic. Birds were captured with Japanese mist nets (#3, EBBA Net Committee, Bryn Athyn, PA), and all captures were examined for ectoparasites. Ticks were removed with forceps and preserved in 70% ethanol for later identification and testing. Birds were then banded and released unharmed. Host-seeking larvae also were collected by drag sampling at the site to further evaluate the potential for transovarial transmission of the HGE agent. To ensure that all larvae were not from a single egg mass, no more than 25 specimens were collected from a single drag.

A total of 123 larval *I. scapularis* were collected from birds captured in May 1993 (one American robin [*Turdus migratorius*] hosting two larvae), July 1997 (one American robin hosting 31 larvae), and August 1997 (two veerys [*Catharus fuscescens*] hosting 10 and 42 larvae, respectively; one wood thrush [*Hylocichla mustelina*] hosting 19 larvae; and one rose-breasted grosbeak [*Pheucticus ludovicianus*] hosting 19 larvae). Additionally, 10 *I. scapularis* nymphs were collected from five of the six birds: 5 and 2 nymphs, respectively, from the robins and a nymph each from the veerys and grosbeak.

Tick infection with *A. phagocytophilum* was determined by polymerase chain reaction (PCR) amplification of a 16S rDNA-associated DNA region specific for this organism. Nymphal ticks were tested individually, and larval ticks were analyzed in pools of five and six individuals; all ticks in a pool were from a single bird. Ticks were minced with a sterile 18-gauge needle in sample buffer provided in the Isoquick DNA extraction kit (ORCA Research, Bothell, WA); DNA was isolated from samples according to the manufacturer’s directions, as described (11). Strict procedures to prevent cross-contamination or carryover of amplified products were employed throughout, as described (11). For *A. phagocytophilum*, a 537-bp PCR product was obtained in a nested PCR reaction protocol employing primers 16S UniF/23S UniR (16S UniF 5´-GAAGTCGTAACAAGG-3´ and 23S UniR 5´-GCCAAGGCATCCACC-3´) in the first reaction, and primers 16S NF/23S NR (16S NF 5´-GTAGGTGAACCTGCGG-3´) and 23S NR (5´-CCAGTGTAAAATACTCTTTCC-3´) in the second round. This PCR protocol resulted in amplification of an appropriately sized product from *E***.** *equi* (PRO Synon. *A. phagocytaphilum)* strain MRK, six cultured clinical isolates of *A. phagocytophila* from HGE patients, and 16 field-collected ticks. No PCR product was obtained from either *E. chaffeensis* or *E. canis* as templates (Liveris and Schwartz, unpub. data). Three clones of each PCR product were randomly selected for sequencing in both the forward and reverse directions. DNA sequence data were collected, edited, and compared to known *A. phagocytophilum* sequences on the BLAST server at the National Center for Biotechnology Information (NCBI) web site (available from: URL: http://www.ncbi.nlm.nih.gov). For *B. burgdorferi*, a 941-bp region of the 16S-23S rDNA spacer was used as a target for amplification using a nested PCR procedure (12).

 Of the 25 larval pools, 5 (20%) tested positive for *A. phagocytophilum—*three pools from a single veery and two pools from a robin. This percentage suggests a minimum infection rate of 4.1% (5/123). In contrast, none of 300 field-collected larvae, comprising 30 pools of 10 larvae each, were PCR positive. This difference is significant (Arcsine transformation of percentages and test of equality; t*_s_* = 3.81, p<0.001). PCR products from one of the *A. phagocytophilum–*positive pools obtained from the robin and from two pools from the veery were cloned and sequenced. All sequences were identical except for a single nucleotide change in the product from the veery. The sequence derived from the tick larvae recovered from the robin was identical to those obtained for the MRK strain of *E. equi* and six *A. phagocytophilum* isolates obtained from HGE patients (Liveris and Schwartz, unpub. data).

Three (12%) of the 25 larval pools were positive for *B. burgdorferi* (one pool each from the wood thrush and both robins). In addition, one of the larval pools from a robin was PCR positive for both *A. phagocytophilum* and *B. burgdorferi.* Thus a single robin may have been coinfected with the causative agents of HGE and Lyme disease and transmitted them to feeding ticks. None of the attached nymphs was positive for either agent, suggesting that larval infection resulting from cofeeding did not occur as a result of feeding in proximity to these nymphs.

These data suggest that at least two species of birds, the American robin and the veery, may be reservoirs for *A. phagocytophium* by infecting larval *I. scapularis* as they feed. Both bird species are reservoirs for *B. burgdorferi* (9), and robins appear to be capable of maintaining and transmitting both the agent of HGE and that of Lyme disease concurrently. This phenomenon has been reported for mammal-feeding *I. scapularis* (13) but not previously for bird-feeding ticks.

Although none of the nymphal *I. scapularis* attached to birds concurrently with larvae showed evidence of *A. phagocytophilum* or *B. burgdorferi* infection, larvae may have acquired both agents by cofeeding with nymphs that detached before capture of the birds. Further work is needed to exclude that possibility.

 Despite small sample sizes, relatively high levels of *A. phagocytophilum* transmission occurred in this study. Because *I. scapularis* larvae remain attached for several days while feeding, replete newly infected larvae may be deposited miles from where they first encountered the host. This pattern effectively seeds new sites with potential vectors of two tick-borne pathogens. However, confirmation that birds can serve as reservoirs of *A. phagocytophilum* is needed, preferably through studies of a wide range of avian species. To date, the number of bird species for which adequate numbers have been captured and from which sufficient numbers of ticks have been collected and tested to determine reservoir status, is quite low. Even in cases where sufficient numbers of birds are collected (10), very low tick loads, a host’s immune response, or both may contribute to low infectivity. In particular, additional work should be conducted in areas where Lyme disease and HGE are endemic. By identifying reservoir-competent bird species and understanding movement patterns that include seasonal migrations covering large distances, the spread of *A. phagocytophila* and the resulting risk that may face human residents in new foci can be clarified.
